# Simulation of Laser Profilometer Measurements in the Presence of Speckle Using Perlin Noise

**DOI:** 10.3390/s23177624

**Published:** 2023-09-02

**Authors:** Sara Roos-Hoefgeest, Mario Roos-Hoefgeest, Ignacio Álvarez, Rafael C. González

**Affiliations:** 1 Department of Electrical, Computer Electronics and Systems Engineering, University of Oviedo, 33003 Oviedo, Spain; ialvarez@uniovi.es (I.Á.); rcgonzalez@uniovi.es (R.C.G.); 2 Desarrollo de Soluciones Integrales Plus S.L., 33211 Gijón, Spain; mroos@cinsystems.es

**Keywords:** laser triangulation profilometry, Perlin noise, speckle, surface roughness, simulation, computer vision

## Abstract

In the manufacturing industry, inspection systems play a crucial role in ensuring product quality. High-resolution profilometric sensors have become increasingly popular for inspection due to their ability to provide detailed surface information. However, the development and testing of inspection systems can be costly and time-consuming. This paper presents the development of a simulation of an inspection system using a high-resolution profilometric sensor. A geometrical and noise model is proposed to simulate the readings of any actual profilometric sensor. The model replicates the sensor’s movement on the CAD model of the inspected part. The model incorporates the physical properties of the sensor and combines noise sources from sensor uncertainty and speckle noise induced by the roughness of the material. Our contribution lies in noise modeling. This work proposes a combination of Perlin noise to simulate the speckle noise and Gaussian noise for the uncertainty-related noise. Perlin noise is generated based on the surface roughness parameters of the inspected part. The accuracy of the simulation system is evaluated by comparing the simulated scans with real scans. The results highlight the ability to simulate real scans of different parts, using commercial sensor specifications and the CAD model of the inspected part.

## 1. Introduction

As increasingly higher requirements are demanded from manufactured products, industries rely on inspection systems to guarantee that their products achieve the expected quality level. This is especially relevant in some industrial sectors, such as the aerospace and automotive sectors or precision parts manufacturing. These sectors demand a full inspection of their production that implies the acquisition of metrological information as well as the detection of other possible defects, such as cracks or irregularities on the product surface. This kind of superficial inspection may be carried out at an intermediate stage of the process to discard defective parts as soon as possible.

However, developing inspection systems can be costly and time-consuming, especially when dealing with high-precision sensors and complex geometries. Therefore, there is a need for CAD tools that can facilitate the development of industrial inspection applications [1,2]. These tools can optimize the design and reduce costs by providing initial data for algorithm development and evaluation. These tools allow for a simulation of the inspection process before actually implementing it on the production line. From the simulation data, it is possible to identify potential issues and refine the inspection process, ultimately improving product quality. To be useful, the simulator must accurately replicate the behavior of the inspection system using models that can be easily related to real hardware. Designers will use that tool to test its performance under different conditions to obtain an accurate and cost-effective solution while reducing the development time and costs.

One of the most common methods for product inspection in industrial applications is laser triangulation. Sensors using this technology employ a laser beam and a camera to measure distances and surface profiles. It works by projecting the laser onto a target surface and analyzing the position of the reflected light in the camera image to calculate the distance. The resolution of triangulation laser sensors refers to their ability to detect and measure small changes or variations in distance. It determines the smallest measurable distance increment that the sensor can reliably capture. The resolution is limited by the uncertainties in the measuring process arising from the different sources of noise in the measuring chain. Among these sources, one of the most important is speckle [3].

Speckle is an interference effect caused by the micro topology of the inspected surface, due to the spatial coherence of the illuminating source [3]. In [4], the influence of speckle in the measurements is analyzed. When lasers are used as light sources on surfaces that are optically rough, speckle can occur due to diffraction. Optically rough surfaces have spatial variations that cause reflected light rays to travel different distances, resulting in phase shifts. These phase-shifted rays can either reinforce or cancel each other out, leading to a pattern of bright and dark areas in the reflected laser light.

Although previous studies have investigated the simulation of laser triangulation sensors for scanning parts, focusing on the geometrical model of the sensor, the impact of the roughness of the scanned material is not usually considered. For example, the work in [5] presents the simulation of scanning laser triangulation sensors for design and evaluation purposes. Based on the CAD files of the parts, the simulation allows for the virtual recreation of the camera acquisition, enabling collision and occlusion detection for complete and effective digitization. In [6], Abu-Nabah et al. proposed a method to validate new laser triangulation designs or commercial ones before their use in the oil and gas industry. They used the commercial animation software Autodesk® 3ds Max® 2017 to create a virtual model of the sensor and generate a synthetic image that is analyzed later on to obtain the depth profile. Using this environment, they were able to check whether the sensor would achieve the required precision and measurement ranges that are needed to retrieve the geometry of weld seams and from that model predict the expected life of the welded product.

Simulation using CAD models can generate measurements that may differ from actual sensor readings because they often overlook the effect of speckle. Just considering the geometrical configuration of the system may produce an accurate ideal measurement, but it will discard the main source of uncertainty, thus limiting the advantages of simulations. It is especially important to consider this discrepancy in applications like surface inspection and defect detection. In this kind of application, the main objective is to assess if differences in surface measurements are relevant and represent a defect or not, for example, an impact mark or a superficial crack. To effectively train algorithms for defect detection and classification, in particular, those used in machine learning or artificial intelligence, it is imperative to include the effects of the main noise sources affecting sensor readings in the simulation models. Ignoring the effect of speckle in simulations can introduce biases and inaccuracies that will compromise the reliability of algorithms in real-world situations.

Speckle simulation has been addressed in some previous works. In [7], Mohammadikaji et al. analyzed the different sources of uncertainty associated with the process of 3D measuring using laser triangulation profilometry. One of the sources of uncertainty that they considered was associated with the localization of the laser line in the image. The accuracy of these values is affected by the presence of speckle combined with quantization effects, sensor noise, sampling, and interpolation methods. They proposed modeling all these effects as two independent zero-mean Gaussian random variables that sum up to the x and y coordinates of the laser line position.

The same research group proposed in [1] a method to obtain realistic simulated images produced by a laser triangulation profilometer. Their objective was to develop a tool for the evaluation and planning of such measurement systems during the development phase. Their model included the simulation of the optical components as well as wave optics effects. They modeled speckle as a diffraction effect caused by a rough surface capable of creating all phase differences. They computed the intensity due to coherent light sources in the Fourier domain, as the product of the estimated amplitude transfer function and the phase of the wave field. As an input to their simulations, they used the CAD model of the object to be measured as well as its bidirectional reflectance distribution function with the corresponding roughness profile. A main drawback was the computational burden of the process. In their work, they stated that rendering an image took about 10 h, using eight cores running at 3.7 GHz.

Csencsics et al. [8] proposed a different approach to simulate speckle. In their simulation, they define all the different elements that form the measuring system, including the geometry, the laser source characteristics, the measured surface, and the imaging system. Then, they approximate the laser spot by a certain number of point sources within the spot diameter, randomly distributed in the z-direction according to the expected surface roughness. For each point source, a given number of rays evenly distributed over a preset solid angle were ray-traced. The solid angle is selected to match the size of the imaging lens. To compute the intensity at each detector point, they combined the different rays arriving at a point according to their phase at that point. They were able to obtain realistic speckle patterns, and they used these results to propose methods to reduce speckle-induced uncertainty.

Other authors have used complex ray tracing methods to model laser triangulation profilometers. In [9], Beermann et al. proposed a simulation model for laser triangulation measurement in an inhomogeneous refractive index field. The simulation setup is performed with a virtual camera and a multistep ray tracing optimization to model a complete triangulation process. The authors explore the benefits of the optical inspection of hot workpieces in multistage forming and the challenges posed by inhomogeneous refractive index fields. The study presents a simulation approach that predicts measurement deviations in such conditions and suggests the design of compensation routines to improve measurement accuracy.

Physically based simulation methods can be very useful when the objective is to design and validate new sensors. As more steps are physically modeled, it is possible to gain more insight into how the sensor performs and the bottlenecks to achieve the desired performance. It may be possible to measure the impact of each component in the final result. Nevertheless, such insight information is useless when the designers want to use an off-the-shelf sensor. In this case, the sensor is a black box that provides depth information. The designer will receive from the camera an array of depth values, where each row corresponds to a single line scan. This information must be processed to extract higher-level information, such as a geometric model or a decision about the presence of defects in the inspected part.

In this work, a proposal to directly simulate the depth measures produced by an off-the-self laser triangulation profilometric sensor is presented. A simple geometrical model provides a base depth value and allows for the simulation of a complete scanning of a part described by its CAD model and its relative position with respect to the sensor during the process. A Gaussian noise model is added to the geometrical depth value to take into account the sensor’s depth resolution and precision. The main contribution of this work is to propose the use of Perlin noise [10,11] to model the effect of all the different physical noise sources present in the scanning process, especially the uncertainty due to speckle. This enables the development of a realistic simulation of the sensor that captures its characteristics and the effect of noise sources. This enhances the effectiveness of the simulation for developing, assessing, and optimizing the algorithms that must analyze this information.

Perlin noise is a type of procedural lattice gradient noise originally used in computer graphics to create procedural textures. It has been used both in films to add realism to computer-generated images and in video games to create terrains procedurally. In the computer graphics community, the term noise is used to refer to a random number generator, while the term procedural refers to the fact that the random value at a given point is computed using an algorithm. The speckle effect is to add a random value to the ground truth one. Instead of simulating the physical process that produces speckle and that will affect the image captured by the sensor camera, we use this well-known procedural noise to compute a random value to be added to any point in the depth image returned by an ideal sensor. A method to tune the parameters governing the Perlin noise algorithm is also described. A good survey of procedural noise functions used in computer graphics can be found in [12,13]

There are some works describing the use of Perlin noise to simulate surfaces. Li et al. [14] proposed a method to simulate the reflection and refraction of water surfaces using Perlin noise and ray tracing. It uses multioctave Perlin noise to construct a random height field of the water surface. Acosta et al. [2] applied Perlin noise to simulate oxide textures for developing an image-processing model for rust detection. They simulate 2D color images with a wide range of possible rusted textures, using the Perlin Noise to simulate only the visual aspect of the surface. Other related works are the one in [15], where the authors use Perlin noise to model the material micro-structure, or the one in [16], which proposes a method to enhance the photogrammetric 3D reconstruction performance of featureless surfaces using noise-function-based patterns. In this work, Perlin noise is one of the noise functions used.

As the final objective is to simulate an off-the-shelf sensor, the information provided by the manufacturer is used to build a the geometrical model. The surface geometry of the part to be inspected is modeled using an STL (Standard Tessellation Language) CAD model. STL is a file format commonly used in the manufacturing industry to provide a digital representation of a 3D object surface. To complete the simulation, the trajectory followed by the sensor with respect to the part surface is used to complete the scanning process. Once the simulation is finished, a set of simulated profiles, arranged as a bidimensional array, is obtained. This is the most typical format in which commercial sensors deliver their measurements to the final user.

## 2. Materials and Methods

Our goal is to achieve a realistic simulation of the readings provided by an off-the-shelf laser profilometric sensor. This should include the effect of speckle, as it is the most relevant source of noise affecting the measurement process. It is a distinct form of noise that occurs as a result of light scattering interference on rough surfaces. In particular, laser triangulation profilometric sensors experience speckle noise due to the irregularities in the surface roughness of the object being scanned. These irregularities introduce fluctuations in the phase and amplitude of the reflected light. The result is the recognizable speckle pattern in the raw image of the laser line. It presents a granular pattern that causes an error in the estimation of the position of its center point. This error is therefore propagated to the estimation of the distance to the object.

This work presents a simulation model that comprises three components: a geometric scene model and two noise models. One accounts for the influence of speckle and the other consider the sensor’s accuracy:

(1) $$\hat{r}\left(x,y\right)=d\left(x,y\right)+PN\left(x,y\right)+P_{G}\left(0,\sigma \right)$$

where $\hat{r}\left(x,y\right)$ represents the global simulated reading, d(x,y) is the distance provided by a geometric model of the scanning, PN(x,y) represents the effect of speckle in the reading, $P_{G}\left(0,\sigma \right)$ represents the error due to the sensor’s accuracy limits, *x* represents the position along the laser line, and *y* represents the scan line.

### 2.1. Geometrical Model

Laser triangulation is a widely used technique in machine vision and metrology for the 3D scanning of surfaces. This non-contact method involves the use of a laser beam and a camera that is directed toward the inspection target. By adopting a known angular offset between the laser source and the camera sensor, it is possible to measure the distance between the surface of the object and the camera using trigonometric principles.

**Figure 1:**
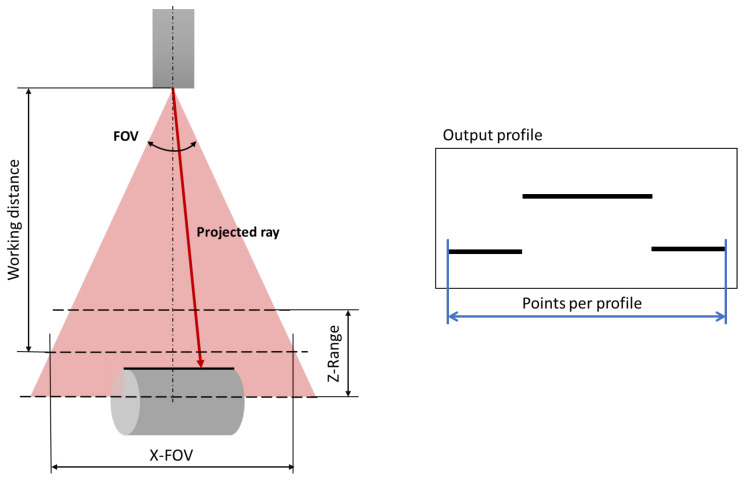
Laser triangulation sensor: key parameters scheme.

The goal is to simulate the readings provided by a commercial laser triangulation profilometric sensor, which provides high-accuracy depth measurements over a line. In this work, a simple geometric model of the sensor is proposed. The model is defined by a set of parameters extracted from the sensor datasheet that are shown in Figure 1. The first parameter is the working distance ($W_{d}$), which accounts for the distance from the camera to the center of the depth measurement range. The working distance is the ideal operating point, where the laser is at its sharpest focal point and the reflected spot is in the center of the detector. Then, we consider the Z-Range ($Z_{R}$) value. It defines the difference between the maximum and minimum measurement distances of the sensor. Therefore, the scanned surface must be within the range $W_{d}\pm \frac{Z_{R}}{2}$ to obtain a valid measurement.

The third parameter used is the field of view (FOV) of the camera. The FOV is the laser beam opening angle and it represents the maximum aperture of the sensor and thus the angular limits of the projected beam. It is computed from the X-FOV and the $W_{d}$. The X-FOV is the total length of the measurement line at the working distance. Therefore, a simple trigonometric computation leads to the following:

(2) $$FOV=2\arctan\left(\frac{X-\mathrm{FOV}}{2W_{d}}\right)$$

The parameter points per profile determine the number of points to be measured in each profile, thus defining the number of rays to be projected in each acquisition. Those rays are evenly distributed within the FOV.

A single profile is not enough to analyze the quality of a surface. It is necessary to scan several profiles and join them to obtain a 3D image of the same. To complete the simulation, the relative trajectory followed by the sensor with respect to the object is replicated. The trajectory is defined by a sequence of sensor pose values and the sensor speed between two consecutive poses. The intermediate position for each individual profile simulation is determined using the maximum profile speed in Hz. This parameter is also available in the sensor datasheet and represents the number of full 3D profiles per second provided by the sensor. Its inverse is the time between two consecutive acquisitions. By employing this temporal interval and the sensor’s travel speed, each intermediary pose is computed through linear interpolation.

The 3D model of the inspected piece is loaded as a triangular mesh, which describes a surface by the unit normal and vertices of the triangles. The profile acquired in each measurement corresponds to the intersections between the projected rays and the triangulated 3D model. It is calculated as a line–triangle intersection case, using the Möller–Trumbore ray–triangle intersection algorithm [17].

The output of the simulation system provides the results of the scan in both a 3D point cloud form and a 2D image, where each row of the image corresponds to a profile of the sensor acquisition, so the size of the image is determined by the number of points per profile and the total number of profiles scanned during the acquisition. Each pixel of the image corresponds to the distance measured by the profilometric sensor.

### 2.2. Speckle Noise Model: Perlin Noise

The inspected surface will always present a certain degree of optical roughness that will cause speckle when is illuminated with a coherent light source. This effect is the main source of error in the measurement. We model this effect by adding a Perlin noise term to the value produced by the geometric model.

#### 2.2.1. Perlin Noise

**Figure 2:**
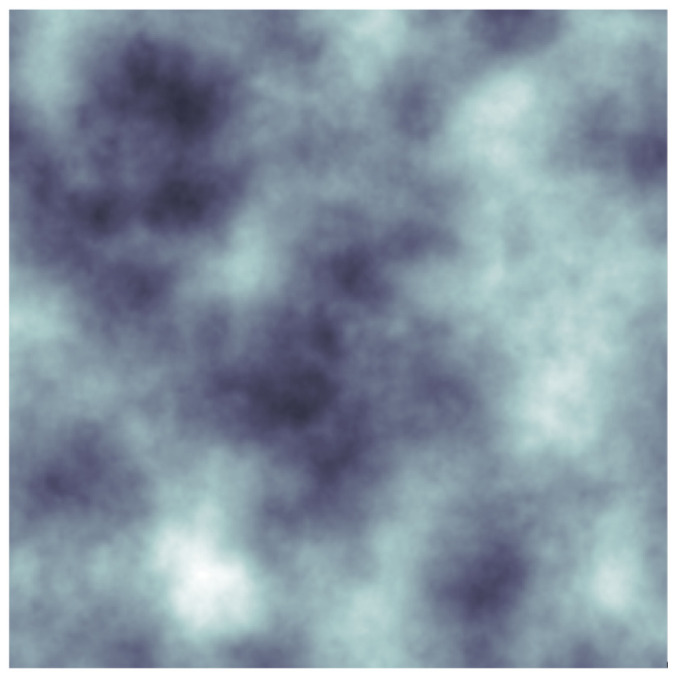
Perlin noise image.

Perlin noise [10] is a type of gradient noise originally used in computer graphics to create procedural textures with a pseudo-random appearance, an example of Perlin noise can be seen in Figure 2. It is defined by the composition of multiple scaled versions of the same noise function, with different frequencies ($f_{m}$) and amplitudes ($A_{m}$), known as octaves. It is described according to Equation (3), where *m* is the *m*-th noise function being added and $n_{octaves}$ is the number of octaves.

(3) $$PN\left(x,y\right)=\sum_{m=1}^{n_{octaves}}A_{m}N_{m}\left(f_{m}x,f_{m}y\right)$$

The level of detail and complexity in the generated texture or pattern is determined by the frequency and amplitude of each octave. The frequency refers to the number of cycles of the noise function within a given unit of space, and higher frequency values result in more rapid variations, producing a more intricate pattern. The frequency of each octave is typically set by multiplying the base frequency ($f_{0}$) by a factor of 2 for each successive octave, expressed as $f_{m}=f_{0}\cdot 2^{m-1}$.

The amplitude determines the range of values that the noise function can output. Higher amplitude values produce more pronounced peaks and valleys, resulting in a more dynamic pattern. The amplitude of each octave is usually determined by a persistence factor *p*, expressed as $A_{m}=A_{0}\cdot p^{m-1}$.

**Figure 3:**
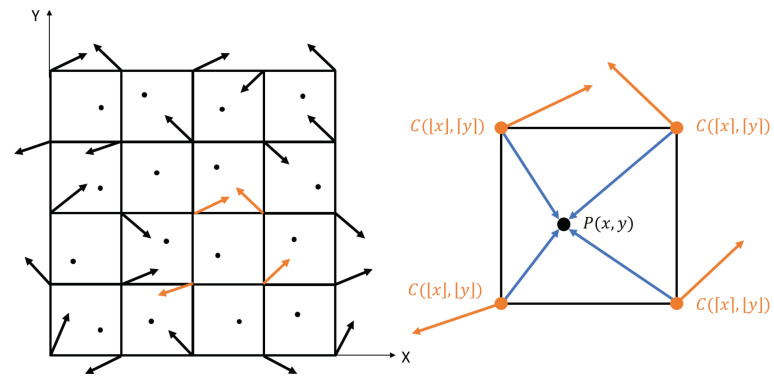
Two-dimensional Perlin noise generation process. Left image shows the 2D grid with the pseudo-random gradient vectors for each control point. Right image represents the process in one input coordinate P(x,y). $C(x_{i},y_{j})$ are the corners of the grid, blue arrows represent the displacement vectors $\vec{d}\left(x_{i},y_{j}\right)$ and orange arrows represent the gradient vectors.

To implement a 2D Perlin noise function, the initial step involves defining a 2D grid of control points. For each control point, a fixed pseudo-random gradient vector of unit length is generated. To compute the value at a point P(x,y), it is necessary to determine the control points lying on the corners of the cell where the point falls $C(x_{i},y_{j})$, where indices (i,j) denote the 4 corners of the cell, and *i* and *j* are either the floor or the ceil of *x* and *y*, respectively, as shown in Figure 3. Then, the position vector of the point P(x,y) with respect to each corner, ($\vec{d}\left(x_{i},y_{j}\right)$), is computed. Equation (4) shows this calculation.

(4) $$\begin{array}{c} \vec{d}\left(x_{i},y_{j}\right)=C\left(x_{i},y_{j}\right)-P\left(x,y\right)\\ i\in \{\lfloor x\rfloor ,\lceil x\rceil \}\\ j\in \{\lfloor y\rfloor ,\lceil y\rceil \} \end{array}$$

For each corner, the dot product between its gradient and displacement vectors is calculated to obtain the influence values $n(x_{i},y_{j})$ for point P(x,y).

(5) $$n_{ij}=\vec{g}\left(x_{i},y_{j}\right)\cdot \vec{d}\left(x_{i},y_{i}\right)$$

The calculation of the 2D Perlin noise function at point P(x,y) is performed according to Equation (7). An interpolation between the 4 dot products is performed to estimate the blending of the noise contribution from the four corners, where $n_{0}$ and $n_{1}$ are the interpolations between $n_{00}$, $n_{10}$ and $n_{01}$, $n_{11}$, respectively (see Equation (6)).

Interpolation is performed using the improved version of the general form of the *smoothstep* function [11], as shown in Equation (8). This function has zero first and second derivatives in the edges, and this ensures a smooth transition between neighboring cells, giving the Perlin noise its characteristic look.

(6) $$\begin{array}{c} n_{0}=n_{00}+smoothstep\left(P_{x}-C_{x_{0}}\right)\cdot \left(n_{10}-n_{00}\right)\\ n_{1}=n_{01}+smoothstep\left(P_{x}-C_{x_{0}}\right)\cdot \left(n_{11}-n_{01}\right) \end{array}$$

(7) $$N\left(x,y\right)=n_{0}+smoothstep\left(P_{y}-Cy_{0}\right)\cdot \left(n_{1}-n_{0}\right)$$

(8) $$smoothstep\left(x\right)=\left\{\begin{array}{cc} 0 & x\leq 0\\ 6x^{5}-15x^{4}+10x^{3} & 0\leq x\leq 1\\ 1 & x\geq 1 \end{array}\right.$$

#### 2.2.2. Perlin Noise Modeling with Roughness Parameters

Based on the properties of Perlin noise, it can be modeled in a way that, by adjusting its parameters appropriately, allows for the simulation of the speckle noise found in real measurements These parameters are the size and the number of control points of the 2D grid; the signal amplitude; the number of octaves; and the persistence factor. In this subsection, we will describe how to select an appropriate set of values to achieve a realistic simulation. Through this approach, a 2D Perlin noise function is estimated and subsequently incorporated into the full scan originating from the geometric model.

Given the variability in the noise in each real measurement, the aim is not to reproduce point-by-point the exact noise patterns but to capture the fundamental characteristics of the noise to allow its simulation. In the presence of speckle, the real readings provided by the laser profilometric sensor look like a rough surface. Although they do not necessarily reflect the true surface roughness of the object, these readings can be adequately characterized using the same parameters that are commonly used to assess the roughness of materials. Roughness parameter values can be obtained through analysis of actual measurements or through pre-existing knowledge of the type of material being scanned. Using these parameters enables the generation of Perlin noise that replicates the noise introduced by speckle.

Surface roughness is a feature of the surface texture. It measures the variation between the actual surface and its ideal form in the direction of the normal vector. Roughness values can either be calculated on a profile (line) or on a surface (area). We consider the parameters profile by profile and generalize them to the surface by taking the average of the values of all the profiles. There are many different parameters to characterize roughness [18]. The most important parameters are the amplitude ones, which characterize the surface based on the vertical deviations of the roughness profile from the mean line.

**Figure 4:**
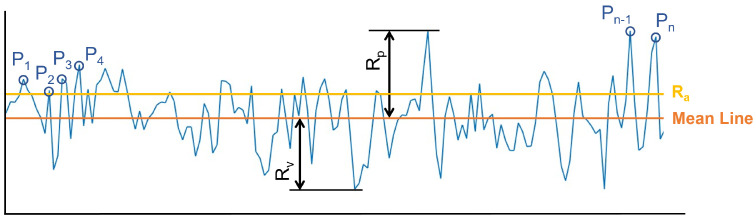
Common roughness parameters in a profile. $P_{i}$ are all the peaks in the profile.

The most common parameter to define roughness is the arithmetic average height ($R_{a}$). It is defined as the arithmetic average of the profile height deviation from the mean line. Other common parameters are the $R_{q}$ (root mean square roughness), which represents the standard deviation of the distribution of surface heights; the $R_{p}$ (maximum height of peaks); and the $R_{v}$ (maximum depth of valleys), as well as the parameters $R_{pm}$ and $R_{vm}$, which measure the mean of the height of the 10 highest peaks and the depth of the 10 lowest valleys, respectively. Figure 4 shows the most important parameters.

Another common parameter is the number of peaks in the profile $P_{c}$. This parameter calculates the number of peaks of the profile per unit length, given in peaks per millimeter. Peaks are only counted when the distance between the current peak and the previous one is greater than 10% of the maximum height of the profile.

After analyzing the most common roughness parameters, those that align most effectively with the Perlin noise model can be determined. Equation (3), which illustrates the Perlin noise generation algorithm involving multiple scaled versions of a single noise function, reveals the critical parameters that require adjustment to correspond with the roughness parameters from the actual measurements. Subsequently, the chosen roughness parameters for modeling Perlin noise are outlined, along with the reason behind these decisions.

First, to generate the 2D Perlin noise function ($N_{m}$), as explained in the previous section, we need to adjust the size of the grid and the number of control points. The grid size is set to match the size of the resulting scan in pixels. This corresponds to the number of points per profile (ppf) and the number of scanned profiles ($n_{profiles}$). The number of control points per dimension is then determined based on $P_{c}$, which allows us to characterize different surface types. We consider that $P_{c}$ provides a reliable approximation of the number of intersections with 0 that the first octave of Perlin noise will have, which corresponds to the number of control points. This parameter is selected because it is more robust than calculating the number of intersections with the mean line of the signal. The number of peaks per mm is estimated by knowing the pixel/mm ratio of a real scan in each direction.

In Perlin noise generation, the frequency represents the number of cycles between two adjacent control points. By aligning the number of control points in the grid with the number of peaks in the desired surface roughness, we can set the frequency to 1. This configuration ensures that there is precisely one complete cycle of the noise pattern between adjacent control points, simulating the spacing between two peaks of the roughness in the resulting noise.

The amplitude is set as the maximum value between $R_{pm}$ and $R_{vm}$. So, the maximum Perlin amplitude corresponds to the highest value of the 10 sharpest peaks or valleys. In this way, the influence of possible outliers is minimized. The other parameters mentioned above will be used to give an estimate of the similarity between the real and simulated samples.

Experimentally, we have found that a number of octaves equal to 4 and a persistence of 0.5 provide satisfactory results, allowing us to add levels of detail to the noise. Increasing the number of octaves does not produce significant changes in the result. For octave five and above, their amplitude is no longer in the measurement range of the sensor and therefore it is no longer detectable.

Substituting the parameters of Equation (3) as explained in this section, Equation (9) is obtained. This equation presents our proposed model to simulate the speckle of the real measurements from Perlin noise. Remember that the amplitude of each octave is determined by the persistence factor as $A_{m}=A\cdot p^{m}$ and the frequency is typically set by multiplying the base frequency by a factor of 2 for each successive octave. $N_{m}$ is the 2D Perlin noise function for each octave, with a grid size to match the size of the resulting scan in pixels and with a number of control points per dimension determined based on $P_{c}$ and the dimensions of the scan.

(9) $$\begin{array}{cc} PN(x,y) & =\sum_{m=1}^{4}\left(0.5^{m-1}\cdot \max\left(R_{pm},R_{vm}\right)\cdot N_{m}\left(2^{m-1}x,2^{m-1}y\right)\right),\\ \; & \forall x\in \left[0,ppf\right],\forall y\in \left[0,n_{profiles}\right] \end{array}$$

### 2.3. Sensor Uncertainty Model: Gaussian Noise

To complete our simulation model, consideration is given to an additional set of sensor parameters: resolution Z ($\Delta_{Z}$) and repeatability ($\sigma_{rep}$). The first one is related to the minimum change in depth that can be reliably detected by the sensor. The second one is the standard deviation of the readings provided by the sensor when repeating the measurement under exactly the same circumstances. The sensor resolution is modeled as a uniform distribution in the range $\left[-\frac{\Delta_{Z}}{2},\frac{\Delta_{Z}}{2}\right]$. The model for the repeatability error is a zero-mean Gaussian distribution with a standard deviation equal to the repeatability parameter. In addition, we consider that both variables are independent, so we model their combined effect as a zero-mean Gaussian with a standard deviation:

(10) $$\sigma =\sqrt{\left(\frac{\Delta_{Z}^{2}}{12}+\sigma_{rep}^{2}\right)}$$

To include the effect of this sensor uncertainty, we add to each measurement a value drawn from a zero-mean Gaussian with standard deviation σ. The final value is trimmed according to the $\Delta_{Z}$ parameter.

## 3. Results

This section presents the results of the laser triangulation profilometry simulator, which incorporates the geometrical and noise model. The algorithms have been implemented using the C++ running on the Ubuntu 20.04 LTS operating system. The open-source Visualization Toolkit software (VTK), version 9.0.0, [19] is used to manipulate the 3D model and simulate the inspection system, and MATLAB 2022b is used to process and analyze the results.

Different experiments are performed to test the reliability of the developed algorithms. Specifically, we replicated real measurements of three different steel products: bearing caps, car doors, and heavy steel plates. These products have different geometries, and, although they are made of the same material, they are manufactured using different processes, resulting in varying surface characteristics.

The results obtained are analyzed by comparing simulated scans with real ones. It is worth noting that quantifying the accuracy of the noise simulation presents significant challenges. The variability introduced by noise complicates the task of quantifying the reliability of the simulation. However, a quantitative analysis of the similarity between simulation and reality can be performed by comparing the distributions of the scans and their surface roughness parameters. Also, a visual comparison of scan appearances will be conducted, considering both individual profiles and the broader scan area.

### 3.1. Geometrical Model

Any commercial profilometric sensor can be simulated by modifying its parameters as needed. In these experiments, the parameters have been tuned to match those of the real inspection system. The actual profilometric sensor used in these experiments is the AT-C5-4090CS30-495 model from Automation Technology. The main parameters extracted from its datasheet [20] are listed in Table 1. Also, the travel speed of the sensor is set as 0.1 m/s.

**Figure 5:**
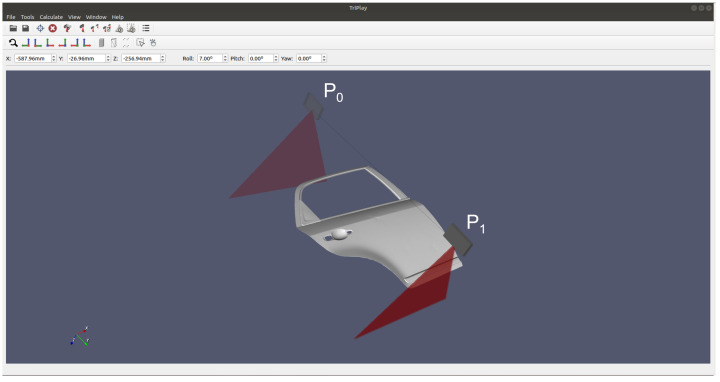
Interface of the developed simulator. The CAD model of a loaded car door and a scan path between the marked points $P_{0}$ and $P_{1}$ is shown.

**Figure 6:**
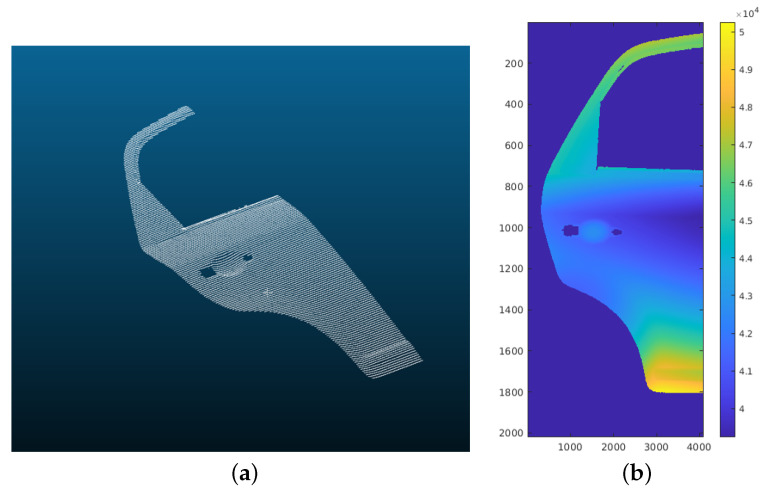
Result obtained during the simulation of a scan of the section of the CAD model shown in Figure 5: (**a**) result in point cloud form; (**b**) result as 2D image. Each row corresponds to a scan profile, so the size of the image is determined by the total number of profiles scanned during the acquisition and the number of points per profile. (Size: 2000 × 4096.)

A 3D model of any part can be loaded as an STL file. The interface of our simulator, with the modeled profilometric sensor, can be seen in Figure 5, where a 3D model of a car door is loaded. Also, the inspection trajectories can be configured to simulate different scans. In this case, it is a straight line between the 2 points $P_{0}$ and $P_{1}$. The travel speed of the scan is set to match the real system. The result of this scan can be seen in Figure 6, as a point cloud and as a 2D image. The size of the image is the total number of profiles by the number of points per profile. The total number of profiles is determined by the travel speed sensor and the scan distance.

### 3.2. Noise Model

In this work, two different noises are presented: the uncertainty of the sensor and the speckle noise. The noise introduced by the uncertainty of the sensor is modeled as a Gaussian noise with an amplitude determined by the resolution of the measurement. The sensor resolution on the *z*-axis, according to the datasheet, is 3.8 μm. However, it is important to note that, based on prior experience and evidence, a more realistic value for the resolution is approximately 15 μm.

Noise introduced in the form of speckle is modeled as Perlin noise. The characteristics of this noise will depend on the material and the manufacturing process of the product. This model can be generalized from the $R_{pm}$, $R_{vm}$, and $P_{c}$ values of each surface.

**Figure 7:**
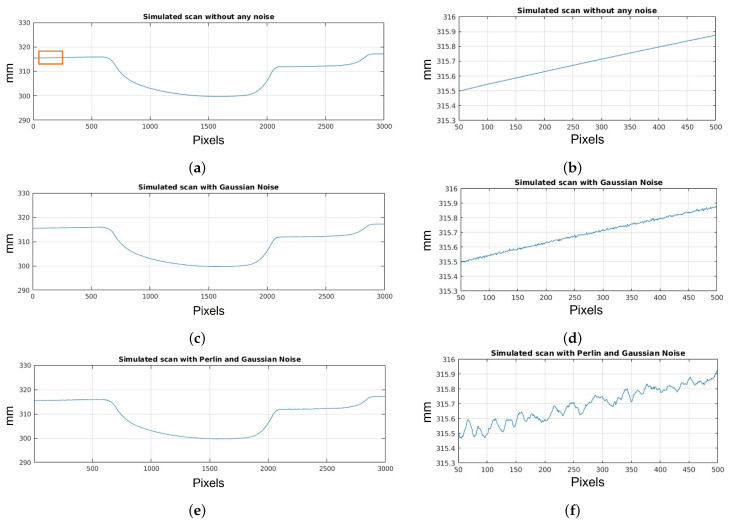
Noise simulation results. Images on the left show the complete profile and those on the right show a zoom of the section outlined in orange: (**a**,**b**) simulation profile before adding any noise; (**c**,**d**) simulation profile after Gaussian noise to simulate sensor uncertainty; (**e**,**f**) simulation profile after Perlin and Gaussian noise.

In Figure 7, a profile extracted from the scan shown in Figure 6 can be observed, specifically from the door handle area. The figures on the left provide a more comprehensive view of the profile, while those on the right are zoomed in on a specific part of it. Figure 7a,b demonstrate that the scan without the addition of noise yields an ideal profile without any alterations. However, this is not desirable for replicating real scans. In Figure 7c,d, Gaussian noise is introduced to simulate the measurement noise of the sensor. The magnitude of this noise depends on the characteristics of the sensor being replicated, which in this case is 15 μm, as previously stated. Also, Perlin noise with an amplitude of 80 μm is included. It should be noted that this is a larger value than the actual roughness of the door material. However, it was calculated this way to see the differences between Gaussian noise and Perlin noise more clearly, only for this particular experiment. Figure 7e,f display the final simulated profile after incorporating Gaussian noise and Perlin noise. It is evident that the Perlin noise has a higher amplitude than the Gaussian noise, as mentioned above. This depends on the specific experiment, taking into account the sensor’s resolution and the surface characteristics of the inspected part.

**Figure 8:**
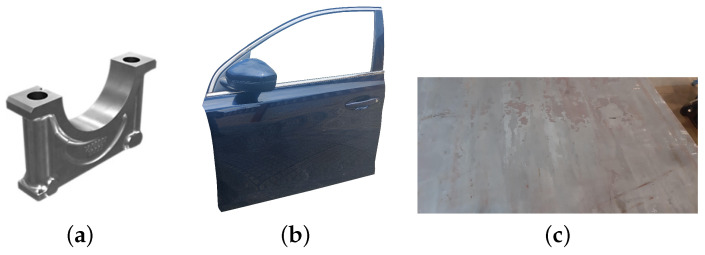
(**a**) Bearing cap (image obtained from [21]). (**b**) Car door. (**c**) Heavy steel plate.

In the following, we will present the results obtained for the simulation of scans of three steel products: bearing caps, car doors and heavy steel plates. Each has a different manufacturing method: bearing caps are manufactured by casting, doors by stamping, and plates by rolling. Figure 8 shows an example of these types of products.

Direct comparison between real and simulated scans is impossible, as noise patterns have a random component and vary with each measurement. Also, our objective is to model realistic measurements rather than replicate specific ones, simulating realistic scans of different products using any laser triangulation sensor. However, a comparison between the roughness parameters of real and simulated scans will be conducted in order to quantify the degree of similarity between scans. Also, their visual appearance will be evaluated, both at the profile and surface levels. In these experiments, scans of more or less flat areas are compared to facilitate the analysis of the results without affecting the shape of the part itself.

To emphasize the importance of simulating the noise introduced by the uncertainty and the noise introduced by the speckle, the same scan will be analyzed, on the one hand, with only the Perlin noise, and, on the other hand, with the full noise model. The roughness parameters of the simulations are estimated in order to compare them with the real sample, in particular, the parameters $R_{a}$, $R_{q}$, $R_{p}$, $R_{v}$, $R_{pm}$, and $R_{vm}$. These parameters are computed as the average of the values calculated for each profile.

Also, we compare the similarity between the real scan distribution and the simulated one using the two-sample Kolmogorov–Smirnov test [22]. It is a non-parametric hypothesis test that evaluates the difference between the CDFs (cumulative distribution functions) of the distributions of the two sample data vectors over the range of x in each dataset. The null hypothesis is that both samples come from the same distribution. A critical value for a 5% significance level is used, so the null hypothesis is rejected if the maximum absolute difference between the CDFs of the two samples is greater than 0.05.

#### 3.2.1. Bearing Cap

**Figure 9:**
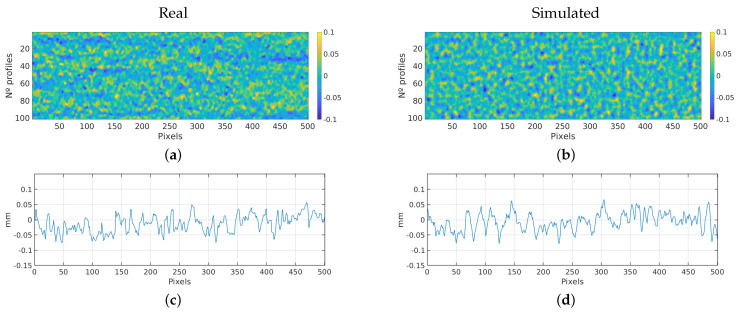
Bearing cap experiment: Comparison between real and simulated scan. (**a**) Real 2D scan image. (**b**) Simulated 2D scan image. (**c**) Real scan profile. (**d**) Simulated scan profile.

This section presents the results obtained during the simulation of scans over a bearing cap. Figure 9 shows the comparison between the results of a simulation scan and a real one. It presents a profile and a 2D image of the obtained scan. It is possible to visually verify the similarity between simulated and real ones. The displayed areas correspond to a portion of the entire scan performed with a sensor of a 4096 pixels per profile. More precisely, it is an area of 100 profiles by 501 pixels, that represents an area of 22.5 × 22.5 mm. A section of the area is presented to offer a clearer view of the noise details and facilitate a more comprehensive analysis, also to preserve the confidentiality of the data.

Table 2 shows the estimated parameters obtained from the analysis of real scans, and compares them with the parameters obtained from the simulation results. Analyzing the table, we observe that the roughness parameters of real and simulated samples are very similar, moving in the same measurement range, with errors around 5%.

**Figure 10:**
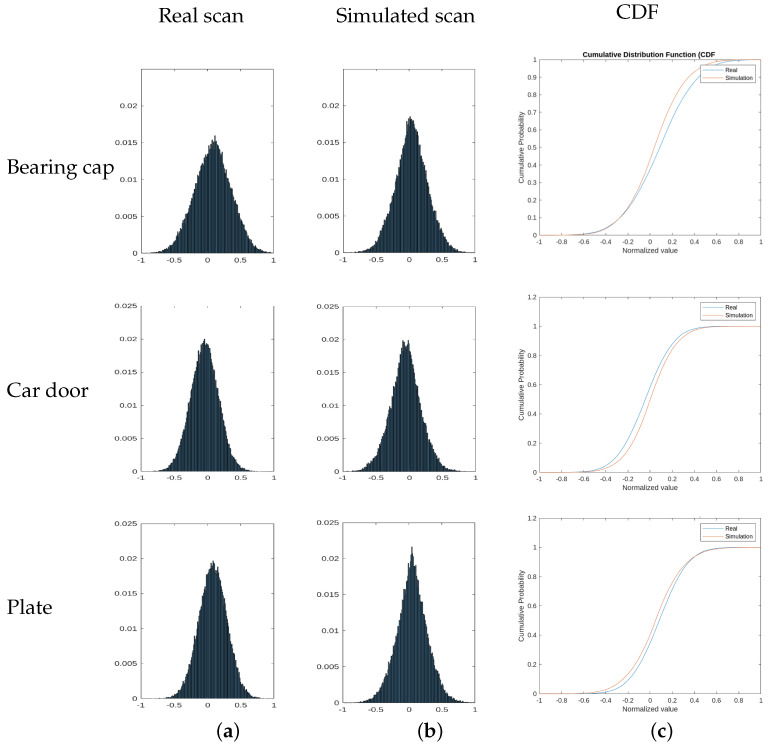
Comparison of distributions: (**a**,**b**) normalized histogram of each of the captures (real and simulated) in the three experiments; (**c**) comparison of the CFDs of the captures.

First row of Figure 10 shows the normalized histogram of real and simulated scan distributions for this experiments. It can be seen that they have similar distributions. After applying the Two-sample Kolmogorov-Smirnov Test, the result is that the null hypothesis is true and, therefore, real and simulated samples come from the same distribution. In Figure 10c the comparison between the cumulative distribution functions is shown.

#### 3.2.2. Car Door

**Figure 11:**
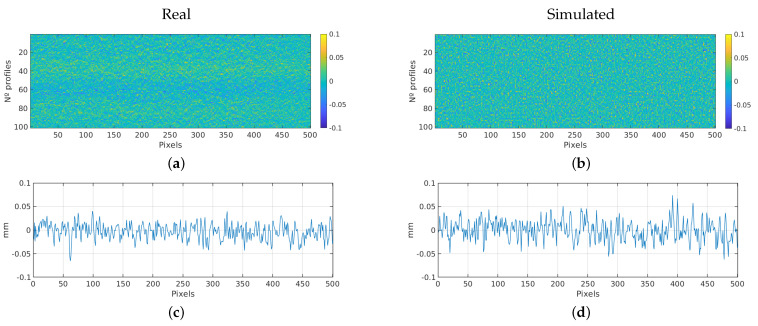
Car door experiment: Comparison between real and simulated scan. (**a**) Real 2D scan image. (**b**) Simulated 2D scan image. (**c**) Real scan profile. (**d**) Simulated scan profile.

This section presents the results of the simulation measurements on a car door. Analogous to the previous section, Table 3 presents a comparative between the roughness parameters of the simulated and real scan. Figure 11 shows the 2D image of the obtained scans. As the previous experiment, it is an area of 100 profiles by 501 pixels, also corresponding to 22.5 × 22.5 mm.

The differences in roughness parameters between the real and simulated scans of the car door surface are relatively small, with percentage errors ranging from 2.03% to 6.28%. This indicates a good agreement between the simulated results and the actual measurements. The simulation accurately captures the essential roughness characteristics of the car door surface, further validating the reliability of the simulation model.

In this case, it can be seen that the roughness values are lower than in the previous experiment. This is due to the characteristics of this particular surface. Both are steel materials, but the ingot is manufactured by casting and the car door by stamping. In general, stamping tends to result in lower surface roughness compared to steel casting. This is because stamping involves shaping the metal through controlled pressure and force, resulting in a smoother and more uniform surface. On the other hand, casting involves pouring molten metal into a mold, which can lead to surface irregularities and textures due to the solidification process.

The Two-sample Kolmogorov-Smirnov Test also confirms that the null hypothesis holds, indicating that both real and simulated samples originate from the same distribution. Figure 10 presents the histograms and the cumulative distribution functions.

#### 3.2.3. Heavy Steel Plate

**Figure 12:**
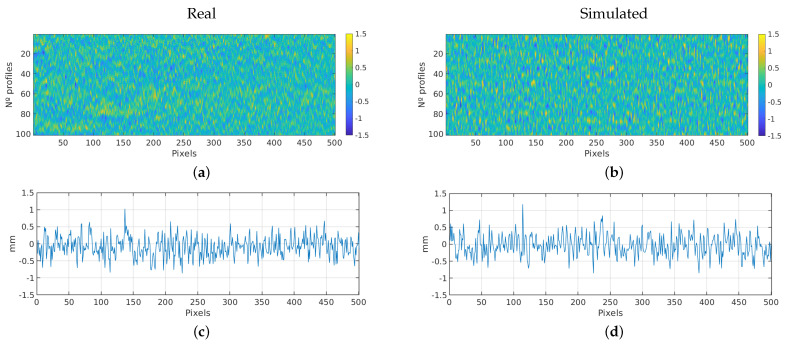
Plate experiment: comparison between real and simulated scans. (**a**) Real 2D scan image. (**b**) Simulated 2D scan image. (**c**) Real scan profile. (**d**) Simulated scan profile.

This section focuses on the simulation scans on a heavy steel plate. The roughness parameters of the simulated and real scans are compared in Table 4. Additionally, Figure 12 showcases a 2D image of the acquired scans, covering an area of 100 profiles with 501 pixels. In this case, the scanning parameters were different and cover an area of 20 × 40 mm.

As in previous experiments, the comparison between real and simulated scans shows that the roughness parameters generally align closely, with small percentage differences, around 4%. Overall, the similarity between the real and simulated roughness parameters indicates that the simulation accurately captures the characteristics of the heavy steel plate’s surface.

As in the previous experiments, we can confirm that both real and simulated samples have similar distributions using the two-sample Kolmogorov–Smirnov test. Figure 10 presents the histograms and the cumulative distribution functions.

Compared to the bearing cap and car door, heavy steel plates typically have higher surface roughness parameters. This is mainly due to the presence of scale or surface oxides, which can contribute to a rougher surface texture and irregularities.

## 4. Discussion

This work proposes a geometrical and noise model for simulating the readings of a commercial laser triangulation profilometric sensor, incorporating the sensor’s physical properties and noise sources. The model accurately reproduces the geometrical characteristics of the scanned surface, as well as the noise patterns caused by speckle and sensor uncertainty. Validation experiments demonstrated a high degree of similarity between the generated scans and real sensor scans. Our research provides satisfactory results, enabling a realistic simulation of laser triangulation scans.

CAD models in STL format are used because is one of the most widely used files to represent the geometry of industrial parts. It stores data as a triangular mesh, which describes a surface by the unit normal and vertices of the triangles. One of the disadvantages is that the resolution of the sensor is higher than the triangular mesh. So, when we simulate the measurement of a profilometric sensor from a CAD model, a profile composed of small straight segments is obtained, corresponding to each of the faces of the triangles that make up the mesh of the model. This would not be a problem in the case of looking for coarse 3D reconstructions, but it is intended to simulate sensors working with resolutions of a few micrometers.

To avoid this problem and allow the simulation of realistic scans, we introduce the simulation of sensor measurement noise and speckle using a combination of Gaussian noise and Perlin noise. It is difficult to quantify the reliability of the noise simulation. In each real scan, the noise is different, both the noise introduced by the sensor and the speckle. However, we can verify the similarity between simulation and reality in our proposal.

This proposal allows the simulation of any commercial laser triangulation sensor from its datasheet, emulating its measurements, including both the measurement noise due to the limitations of the sensor itself and the noise generated by the surface of the scanned product. We allow a simulation of the noise in a relatively simple way, considering surface roughness parameters, without entering into complicated physical procedures.

We successfully simulated the speckle generated by the roughness of different types of materials, focusing specifically on three steel products manufactured using different fabrication methods. The results of our study highlight the accuracy of our modeling and simulation process for a wide range of surfaces. Comparison between the actual scans and our simulated results for the bearing cap, cab door, and plate surfaces consistently showed similar roughness characteristics. However, by generalizing the noise model using roughness parameters, we can simulate different surfaces, based on a priori knowledge of the type of material and surface finish or by a simple analysis of roughness parameters from a real scan.

The comparative analysis highlights small differences between the simulated and real scans for the roughness parameters in the three experiments. The small errors for the roughness parameters indicate that the simulation approach effectively captures the general roughness characteristics of the surfaces. Therefore, the results suggest that the current simulation method adequately reproduces the essential roughness features.

Overall, the results presented in this work validate the effectiveness of the simulation approach for reproducing the real scans. The similarity between the real and simulated scans, as observed in both the visual comparison and the quantitative analysis, indicates that the simulation accurately captures the essential roughness characteristics of each surface. The ability to accurately simulate surface roughness opens up possibilities for using simulated scans in different algorithms and applications, offering a cost-effective and efficient alternative to extensive experimental measurements.

The purpose of the proposed model is not to precisely replicate real measurements but rather to enable the simulation of real measurements obtained from commercial sensors. Speckle is an interference effect caused by the microtopology of the inspected surface, due to the spatial coherence of the illuminating source. This noise is simulated using a Perlin noise model, avoiding the complex real-world physical processes that produce it. This allows us to simulate measurements equivalent to actual measurements in a simpler and faster way. However, this limitation prevents us from using the simulator for the creation of new sensors or for other purposes, such as calculating the level of influence of error induced by speckle or other sources. However, this allows us to simulate realistic scans using 3D models that do not provide any roughness information.

Also, this work allows for a test bench to try different data acquisition methods. The most optimal trajectories of the sensors can be searched to scan different types of parts and see how this affects the 3D reconstruction and the identification and measurement of the defects. This is useful to allow the design of the acquisition system prior to its installation on product lines. To ensure measurement accuracy, companies need to build complex systems with expensive high-accuracy sensors on production lines. Also, the design of the acquisition system is performed before knowing the optimal configuration. This leads to increased costs and development time. Therefore, it is important to obtain a system that provides a realistic simulation of the acquisition system and the defects to be inspected.

As part of our future research directions, the focus will be directed towards surface defect detection in manufacturing using profilometric sensors. Many industries still rely on manual inspection due to the limited defect data for developing defect detection algorithms. Collecting authentic defect data is an arduous and costly task, especially during development. In addition, the sporadic nature of defects in industrial processes adds another layer of complexity. To address these problems, future work aims to enable the creation of a synthetic defect database including dimensional data. To this end, work is being conducted to simulate real defects in 3D models. From this, the scanning process of profilometric sensors will be reproduced using the simulator proposed in this paper. It is important to highlight that noise has an impact on measurements, so it is the key to accurately replicating it in the simulated measurements, as explained in this work. Having a bank of defects is crucial, especially when it comes to developing artifical intelligence algorithms effectively.

## 5. Conclusions

This work proposes a simulation model to replicate the readings of a real laser triangulation profilometric sensor, which provides high-accuracy depth measurements over a line. The model is composed of three different components: a geometrical model accounting for the sensor’s physical attributes, a Gaussian noise model simulating sensor uncertainty, and a Perlin noise model mimicking speckle-induced noise.

The presented approach was validated through experiments that compare the 3D scans generated by the proposed model to those provided by a real laser triangulation profilometric sensor. Results found that the simulator is capable of accurately reproducing the geometrical characteristics of the scanned surface, as well as the noise patterns introduced by the material roughness and the sensor itself. The generated scans had a high degree of similarity to the real scans.

The results of the research have provided quite satisfactory results. Overall, the proposed geometrical and noise models allow for the creation of a simulation of an off-the-self sensor that accurately represents the behavior of the real sensor, including the effects of the different noise sources, especially speckle. This simulation model stands as a valuable asset for the design and evaluation of real-world applications, facilitating the assessment of data processing and analysis algorithms before physical system deployment. In this way, better solutions may be achieved while reducing the development time and costs.

## Figures and Tables

**Table 1:** Parameters of the profilometric sensor from its datasheet and travel speed used in these experiments.

Working Distance	400 mm
Z-Range	250 mm
FOV	63.5${}^{\circ }$
Points per profile	4096 pixels
Z resolution	3.8 μm
Profile speed	500 FPS
Travel speed	0.1 m/s

**Table 2:** Bearing Cap: Comparison of roughness parameters between real and simulated scans. Estimated as the average of the parameters calculated for each profile.

Bearing Cap			
	**Real**	**Simulation**	$\epsilon_{r}\left(\%\right)$
$R_{a}$ (mm)	0.0211	0.0208	1.29
$R_{q}$ (mm)	0.0263	0.0263	0.11
$R_{p}$ (mm)	0.0731	0.0764	4.47
$R_{v}$ (mm)	0.0742	0.0790	6.55
$R_{pm}$ (mm)	0.0676	0.0702	3.76
$R_{vm}$ (mm)	0.0700	0.0741	5.85
$P_{c}$ (peaks/mm)	4.36	4.62	5.74

**Table 3:** Car door: Comparison of roughness parameters between real and simulated scans. Estimated as the average of the parameters calculated for each profile.

Car Door			
	**Real**	**Simulation**	$\epsilon_{r}\left(\%\right)$
$R_{a}$ (mm)	0.0134	0.0137	2.03
$R_{q}$ (mm)	0.0169	0.0173	2.47
$R_{p}$ (mm)	0.0523	0.0542	3.65
$R_{v}$ (mm)	0.0522	0.0555	6.28
$R_{pm}$ (mm)	0.0466	0.0482	3.50
$R_{vm}$ (mm)	0.0477	0.0504	5.73
$P_{c}$ (peaks/mm)	5.9446	6.2403	4.97

**Table 4:** Heavy steel plate: Comparison of roughness parameters between real and simulated scans. Estimated as the average of the parameters calculated for each profile.

Heavy Steel Plate			
	**Real**	**Simulation**	$\epsilon_{r}\left(\%\right)$
$R_{a}$ (mm)	0.2484	0.2403	3.27
$R_{q}$ (mm)	0.3118	0.3058	1.94
$R_{p}$ (mm)	0.9397	0.9633	2.52
$R_{v}$ (mm)	0.9834	0.9642	1.95
$R_{pm}$ (mm)	0.8329	0.8550	2.65
$R_{vm}$ (mm)	0.8925	0.8817	1.22
$P_{c}$ (peaks/mm)	6.8758	6.2277	9.43

## Data Availability

The data presented in this study are available from the corresponding authors on request.

## Abbreviations

The following abbreviations are used in this manuscript:

$A_{m}$	Amplitude of Perlin noise function *m*-th octave
$A_{0}$	Perlin noise algorithm base amplitude
CAD	Computer-aided design
CDF	Cumulative distribution function
$C(x_{i},y_{i})$	Cell corners surrounding point (x,y)
d(x,y)	Depth from sensor to object at point (x,y)
$\Delta_{z}$	Sensor depth resolution
$f_{m}$	Frequency of Perlin noise function *m*-th octave
$f_{0}$	Perlin noise algorithm base frequency
FOV	Field of view
n(i,j)	Influence value of corner $C(x_{i},y_{i})$ for point (x,y)
$N_{m}\left(f_{m}x,f_{m}y\right)$	*m*-th noise function component
$n_{octaves}$	Number of terms (octaves) used to build the Perlin noise
$n_{profiles}$	Number of scanned profiles
*p*	Perlin noise algorithm persistence factor
$P_{c}$	Number of peaks in the profile
$P_{G}\left(0,\sigma \right)$	Error due to sensor accuracy limits
PN(x,y)	Perlin noise value at point (x,y)
$\hat{r}\left(x,y\right)$	Simulated sensor reading at point (x,y)
ppf	Points per profile
$R_{a}$	Arithmetic average height
$R_{q}$	Root mean square roughness
$R_{p}$	Maximum height of peaks
$R_{v}$	Maximum depth of valleys
$R_{p}m$	Mean of the height of the 10 highest peaks
$R_{v}m$	Mean of the depth of the 10 lowest valleys
$\sigma_{rep}$	Sensor repeatability
STL	Standard Tessellation Language
$W_{d}$	Sensor working distance
X−FOV	Field of view along the horizontal direction
$Z_{R}$	Sensor Z-Range value
